# Associations of perceived natural and social environments with subjective well-being among Chinese older adults: a cross-sectional study of gender differences

**DOI:** 10.3389/fpsyg.2026.1880057

**Published:** 2026-07-15

**Authors:** Xiong Chengxia, Jin Jing

**Affiliations:** School of Publishing, University of Shanghai for Science and Technology, Shanghai, China

**Keywords:** gender differences, neighborhood interaction, perceived natural environment, perceived social environment, subjective well-being of older adults

## Abstract

**Background:**

Environmental psychology research is characterized by a divergence between two core theoretical orientations. The stress reduction theory posits a direct correlation between perceived natural environment and individual well-being; the social ecology theory, in contrast, maintains that the correlation between perceived social environment and subjective well-being is contingent upon social behavior. However, few studies have systematically compared the differentiated association patterns of these two environment types with older adults’ subjective well-being within a unified analytical framework, and integrated examination of gender heterogeneity in such associations remains inadequate. This gap delays theoretical integration and provides limited evidence for gender-sensitive age-friendly community design.

**Methods:**

This study used data from the 2021 Chinese General Social Survey (CGSS), yielding 2,929 valid participants aged 60 and above. All core environmental variables were assessed through subjective perception rather than objective exposure. Perceived natural environment focused on negative perceived exposures; perceived social environment covered comprehensive evaluations of neighborhood safety, living convenience, neighborhood mutual assistance, and public services. A structural equation model linking perceived natural environment, perceived social environment, neighborhood interaction, and subjective well-being of older adults was specified. Associations were estimated using the WLSMV estimator. To ensure the validity of gender comparisons, measurement invariance tests were conducted. Common method bias was assessed using the latent method factor approach. Gender heterogeneity was examined through multi-group structural equation modeling. In addition, multiple imputation was performed to handle missing data and to test the robustness of the results against potential bias due to incomplete responses.

**Results:**

In the full sample, the total association of perceived social environment with subjective well-being (*β* = 0.261, *p* < 0.001) was significantly stronger than that of perceived natural environment (*β* = 0.140, *p* = 0.006). The association between perceived social environment and subjective well-being consisted of both a direct association and an indirect association involving neighborhood interaction (*β* = 0.081, *p* < 0.001), whereas for perceived natural environment, the indirect association was not significant. Gender heterogeneity analyses further showed that perceived natural environment was positively associated with subjective well-being only among older men (*β* = 0.222, *p* = 0.002) and exhibited only a direct association. Perceived social environment was positively associated with subjective well-being only among older women (*β* = 0.322, *p* < 0.001), and this association included an indirect component involving neighborhood interaction (*β* = 0.073, *p* = 0.005).

**Conclusion:**

This study clarifies the differentiated theoretical patterns of association between perceived natural environment and perceived social environment with older adults’ subjective well-being, confirms the gender-specific divergence in these associations, and provides localized empirical evidence for developing a gender-sensitive, precision-oriented environmental support system for age-friendly communities in China.

## 1 Introduction

With accelerating population aging, the association between the community environment and older adults’ subjective well-being has become an important topic in environmental psychology (Zheng et al., 2025; Nobile et al., 2023). For older adults whose activity space is highly concentrated in their residential neighborhoods, the community is not an abstract spatial backdrop but an everyday life setting in which positive psychological experiences and subjective well-being are attained (He et al., 2024). Compared with the general adult population, older adults have a smaller daily activity radius and greater dependence on the residential community environment (Zheng et al., 2021); thus, their subjective perception of the community environment may be more directly associated with their subjective well-being.

Existing environmental psychology research has separately documented the associations of perceived natural environment and perceived social environment with older adults’ subjective well-being (Zhang et al., 2025; Hegewald et al., 2020; Yang, 2020; Stafford et al., 2011; McElroy et al., 2019); however, the theoretical logic underlying these associations is not consistent (Del Barrio et al., 2021). On the one hand, studies rooted in stress reduction theory tend to regard negative perceptions of the natural environment as environmental stressors: elements such as air pollution, water pollution, and noise pollution are linked to reduced environmental comfort and satisfaction (Zhang et al., 2025; Hegewald et al., 2020) and, in turn, are directly and negatively associated with older adults’ subjective well-being (Yang, 2020). On the other hand, research based on the social ecology perspective indicates that perceived social environment—encompassing neighborhood safety, living convenience, neighborhood mutual assistance, and public services—may be not only directly and positively associated with older adults’ positive life experiences (Stafford et al., 2011) but also indirectly associated with their subjective well-being through daily interaction, social support, and social isolation (McElroy et al., 2019). These two theoretical orientations imply that the logic of association between different types of environmental perception and subjective well-being may differ fundamentally (Gong et al., 2024; Liu et al., 2020). However, most existing studies have focused on a single environmental dimension, making it difficult to clarify the differentiated association patterns of the two within a unified analytical framework. To address this, the present study simultaneously incorporates perceived natural environment and perceived social environment into the analytical framework and systematically examines their associations with older adults’ subjective well-being, thereby remedying the limitation of focusing on only one dimension in current environmental psychology research.

Neighborhood interaction is one of the most common and community-embedded everyday social behaviors among older adults (Wang et al., 2025). Social-ecological systems theory emphasizes that individual well-being is not solely a function of personal characteristics but is embedded in the interplay of external environments, everyday behaviors, and social relationships (Stokols, 1996). For older adults whose daily activity radius is largely confined to the community, favorable perceived community environment may be associated with enhanced neighborhood trust, more frequent daily contact, and greater mutual assistance, which in turn are associated with higher subjective well-being (Wang et al., 2025; Song et al., 2023; Chen and Zhang, 2022). In contrast, whether perceived natural environment is also indirectly associated with subjective well-being through neighborhood interaction remains unknown and requires further empirical examination (Moore et al., 2018). Comparing this divergence in association patterns helps to theoretically distinguish between two explanatory accounts: the direct association of environmental stressors and the behavior-contingent association of perceived social environment.

Furthermore, the associations among environmental perceptions, neighborhood interaction, and subjective well-being may exhibit pronounced gender heterogeneity among older adults (Chen et al., 2022). Older men and older women differ in family roles, daily activity radius, dependence on community resources, and modes of social interaction (Zhao et al., 2023); these differences may correspond to divergent patterns of association between the same type of community environment perception and subjective well-being across genders (Li et al., 2022). Although existing studies have separately examined gender heterogeneity in the associations of perceived natural environment and perceived social environment with older adults’ well-being, few have compared within a single analytical framework whether the associations of the two types of environmental perception with older adults’ subjective well-being differ by gender.

Drawing on the above theoretical tensions and research gaps, this study constructs a comparative framework with two sequences of association: (a) perceived natural environment—neighborhood interaction—subjective well-being, and (b) perceived social environment—neighborhood interaction—subjective well-being. It should be noted that the environmental variables in this study are all subjective evaluations of the community environment provided by older adults, rather than objective environmental data. Using data on perceived environments and subjective well-being from the Chinese General Social Survey, it addresses three research questions: first, it compares the strength of the associations of perceived natural environment and perceived social environment with older adults’ subjective well-being; second, it examines whether neighborhood interaction shows a differentiated indirect association in the link between these two types of environmental perception and subjective well-being; third, it uses multi-group structural equation modeling to test whether the aforementioned association sequences differ significantly between older men and older women. The present study theoretically extends the application of environmental psychology theory within gerontology and, practically, provides localized empirical evidence for gender-sensitive, age-friendly community design.

## 2 Materials and methods

### 2.1 Data source

This study drew data from the 2021 Chinese General Social Survey (CGSS) database. The CGSS is a nationwide, continuous, comprehensive large-scale social survey conducted by the National Survey Research Center at Renmin University of China. It systematically collects data on residents’ living conditions across community, household, and individual levels, and serves as one of the most widely used representative micro-databases in Chinese social science, public health, and urban planning research. The survey adopts a rigorous multi-stage, stratified, and proportionate to size probability sampling method, with the sampling process divided into 5 stages: the first stage uses county-level administrative regions (districts, counties, county-level cities) as the primary sampling units, the second stage is streets/towns, the third stage is communities/residential committees (villages/village committees), the fourth stage is family households, and the fifth stage is randomly selected survey subjects within the households.

The 2021 CGSS covered 28 provinces/autonomous regions/municipalities (excluding Xinjiang Uygur Autonomous Region, Tibet Autonomous Region, Hainan Province, Hong Kong, Macao, and Taiwan), and the full database contained 13,934 valid respondents of all ages. The inclusion criterion for this study was respondents aged 60 years or older at the time of the survey. The exclusion criteria were: missing values on core variables or obvious logical errors in the data (e.g., values outside the predefined scale range). After age-based screening, an initial sample of 4,367 older adults aged 60 and above was obtained. Upon item-by-item examination, 56 cases with clear skip-pattern errors or invalid values were removed, leaving 4,311 cases entering the missing data analysis phase. The overall missing rate for the core variables in this study was 32.1% (1,382/4,311). Although Little’s MCAR test indicated that the missing pattern of the core variables did not significantly deviate from the assumption of missing completely at random (*p* = 0.172), the high missing rate may be associated with subgroups of older adults experiencing physical or cognitive decline, potentially introducing systematic bias (Little, 1988). Therefore, in addition to listwise deletion, multiple imputation (MI) was used as a robustness check to ensure that the findings were not contingent upon the missing data handling method (Austin et al., 2021). Specifically, the supplementary verification using multiple imputation was conducted via the chained equations method (MICE), generating 20 complete datasets. The imputation model included all core variables (perceived natural environment, perceived social environment, neighborhood interaction, subjective well-being) and control variables strongly associated with the probability of missingness. All statistical analyses were performed separately on each of the 20 imputed datasets, and the results were combined using Rubin’s rules. Results showed that the findings based on multiple imputation were highly consistent with those from listwise deletion in direction, magnitude, and statistical significance, indicating that the study’s conclusions were not sensitive to the missing data treatment.

The final analytic sample comprised 2,929 valid cases. The socio-demographic characteristics of the sample were as follows: 1,426 men (48.7%) and 1,503 women (51.3%); age range 60–95 years, with a mean age of approximately 70 years; and educational attainment predominantly at the level of primary school or below (62.3%). The socio-demographic profile of the sample closely matched the sex, age, and educational distribution of the population aged 60 and above in the Seventh National Population Census, demonstrating good representativeness and meeting the sample selection criteria for interdisciplinary research on environmental health and age-friendly design.

### 2.2 Measurement method

#### 2.2.1 Dependent variable: older adults’ subjective well-being

The dependent variable in this study is subjective well-being among older adults, measured using the World Health Organization Five Well-Being Index (WHO-5). The WHO-5 is a globally standardized instrument recommended by the World Health Organization for assessing subjective well-being, with well-established cross-population reliability and validity and particular suitability for large-scale well-being screening in older populations (Topp et al., 2015). Oriented primarily toward positive affect, the scale tends to elicit lower psychological defense and reporting bias; it also features brevity, ease of administration, and broad applicability, though it should not be regarded as a clinical diagnostic tool.

The WHO-5 comprises five core items that comprehensively cover the central dimensions of older adults’ daily psychological experience: (1) I have felt cheerful and in good spirits; (2) I have felt calm and relaxed; (3) I have felt active and vigorous; (4) I woke up feeling fresh and rested; (5) My daily life has been filled with things that interest me. In this study, all items were scored on a 5-point Likert scale ranging from 1 = at no time to 5 = all of the time, with higher scale scores indicating higher subjective well-being. Reliability analysis yielded a Cronbach’s *α* of 0.853, demonstrating good internal consistency.

#### 2.2.2 Independent variables: perceived natural environment and perceived social environment

The core independent variables are perceived natural environment and perceived social environment, both constructed from standardized CGSS 2021 items. Subjective environmental perception was prioritized over objective monitoring data for three main reasons. First, theoretically, objective environment requires subjective cognitive processing before it becomes associated with subjective well-being among older adults; perceived environment is therefore more closely associated with residents’ well-being, an issue of particular relevance for older adults whose activity space is highly confined to the community (Liao et al., 2015). Second, methodologically, objective data at the county or district scale cannot capture microscale community heterogeneity and may produce measurement biases such as high green coverage combined with poor accessibility (Zhang and Zhang, 2017). Third, practically, subjective perception directly reflects the genuine needs of the older population and can provide actionable intervention strategies for precision age-friendly community planning.

Perceived natural environment: In this study, perceived natural environment was operationalized as older adults’ subjective evaluation of the risk of negative natural environment exposure at the community scale. The focus on negative exposure rather than positive elements is motivated by evidence that the association between negative natural environment exposure and older adults’ subjective well-being is generally stronger than the association of positive elements with subjective well-being among older adults (Zhang et al., 2025; Hegewald et al., 2020). Four core dimensions—air pollution, water pollution, noise pollution, and insufficient daylight—were selected to form the scale, and all items were rated on a 5-point Likert scale. All items were reverse coded so that higher scores represent better perceived natural environment quality. The scale demonstrated a Cronbach’s *α* of 0.862, indicating good reliability.

The inclusion of these indicators is justified by the following: they represent the most prevalent environmental concerns in Chinese urban communities; they are the only community-level negative natural environment exposure indicators systematically measured in the CGSS. Regarding the exclusion of objective indicators, on the one hand, objective indicators and subjective perception constitute distinct constructs, and this study focuses on the theoretical sequence of associations involving perception, behavior, and well-being, for which subjective perception serves as the starting point. On the other hand, the CGSS data cannot precisely link individuals to community-scale objective environmental data, and forcibly incorporating county/district-level aggregate indicators would create scale mismatch and measurement bias.

Perceived Social Environment. In this study, perceived social environment was operationalized as older adults’ subjective evaluations of neighborhood safety, shopping convenience, satisfaction with community management, and neighbors’ willingness to help. The selection of these indicators is based on the WHO Global Age-Friendly Cities Guide, which explicitly identifies three dimensions of the social environment—neighborhood social support, community governance efficacy, and public service accessibility—thereby comprehensively covering the core concerns of the older population. All items were rated on a 5-point Likert scale, with higher scores indicating better perceived social environment quality. The scale yielded a Cronbach’s *α* of 0.872, confirming a scientifically sound measurement model.

#### 2.2.3 Mediating variable: neighborhood interaction

The mediating variable in this study is the neighborhood interaction among older adults, which is measured through 4 core items derived from the social interaction module of the CGSS 2021. The items correspond to the original survey questions: “The frequency of entertainment activities with neighbors,” “The frequency of mutual assistance between you and neighbors,” “The frequency of chatting and getting together with neighbors,” and “The frequency of visiting neighbors’ homes.” All items were rated on a 5-point Likert scale, with 1 indicating “never” and 5 indicating “almost every day,” where higher total scores reflect more frequent neighborhood interaction among respondents. Consistent with established measurement practices in gerontological and urban sociology research, the reliability test results demonstrated that the Cronbach’s *α* coefficient of this scale in the current study was 0.840, indicating good internal consistency reliability and supporting the scale’s suitability for measuring neighborhood interaction among Chinese older adults.

#### 2.2.4 Control variables

To account for potential confounding and enhance the robustness and precision of the model estimates, and in line with established practices in research on perceived environment and older adults’ subjective well-being, individual-level sociodemographic characteristics were included as control variables (Frank, 2000). These comprised age, gender, marital status, educational attainment, number of co-residents, self-perceived economic class, and self-rated health. Age and number of co-residents were treated as continuous variables and entered into the model as the respondent’s actual age in years and the number of people living together, respectively. Sex (1 = male, 2 = female) and marital status (1 = currently married, 2 = other) were binary variables. Educational attainment was coded from 1 (“illiterate”) to 7 (“graduate and above”), self-perceived economic class from 1 (lowest) to 10 (highest), and self-rated health from 1 (“very unhealthy”) to 5 (“very healthy”).

### 2.3 Research design

This study was fully conducted using Mplus 8.0 and SPSS 26.0 software for statistical analysis, consistent with standard practice in structural equation modeling (SEM) research (Asparouhov and Muthén, 2009). The data analysis process was divided into four core stages.

#### 2.3.1 Core analysis process

In the first stage, descriptive statistical analysis was conducted to characterize the demographic and sociological characteristics of the sample and the distribution of all observed variables, verifying data normality and dispersion to lay the foundation for subsequent model construction.

In the second stage, Confirmatory Factor Analysis (CFA) was employed to validate the convergent validity, discriminant validity, and overall fit of the latent variable measurement model, a critical prerequisite for robust SEM analysis (Steenkamp and Maydeu-Olivares, 2023).

In the third stage, a structural equation model was specified to systematically examine the association sequences involving perceived natural environment/social environment, neighborhood interaction, and older adults’ subjective well-being. The model was specified such that the two independent variables could have direct associations with subjective well-being and also indirect associations through neighborhood interaction (parallel indirect associations); all control variables were included as exogenous variables to account for their potential associations with the dependent variable. Bias-corrected bootstrapping with 1,000 resamples was used to compute 95% confidence intervals for the path coefficients and indirect associations; a confidence interval that did not contain zero was considered indicative of a statistically significant estimate (Cheung, 2007). Additionally, the results were verified through multiple imputation to ensure that the findings were not sensitive to missing data.

In the fourth stage, multi-group measurement invariance testing was conducted to verify the measurement equivalence of the four latent variables (perceived natural environment, perceived social environment, neighborhood interaction, and subjective well-being) across older men and older women, thereby ensuring the validity of subsequent between-group comparisons of the associations (Fischer and Karl, 2019). On this basis, multi-group SEM was employed to examine the heterogeneity of the core association patterns across gender groups, verifying cross-group stability and the statistical significance of differences in these associations.

#### 2.3.2 Estimation method selection

Given that the observed indicators of the core variables in this study are all ordinal categorical Likert-type variables and the data do not satisfy the strict assumption of multivariate normality, the weighted least squares mean and variance adjusted (WLSMV) estimator was adopted for model estimation. This method is specifically designed for ordinal categorical variables, performs better with non-normally distributed data, and represents the current standard approach for SEM with such variables. In the robustness check, for the multiple imputation datasets, robust maximum likelihood (MLR) estimation was used; this estimator effectively handles non-normal data and provides robust standard errors (Austin et al., 2021).

#### 2.3.3 Common method Bias control

Because all latent variables were derived from cross-sectional self-reports obtained from the same respondents, common method bias (CMB) could potentially inflate the coefficients. To address this concern, both procedural and statistical controls were employed. Procedurally, the questionnaire design incorporated different scale formats, reverse-coded items, and randomized item order to reduce response consistency bias. Statistically, Harman’s single-factor test was first conducted; the unrotated first factor explained only 28.7% of the total variance, which is below the 40% threshold, indicating no serious single-method-factor problem. A more stringent test was then performed using a CFA model that included an unmeasured latent method factor on which all items were allowed to load in addition to their respective substantive factors. The model fit significantly improved after adding the method factor; however, all standardized factor loadings of the observed variables on their substantive latent factors remained above 0.6, and the average variance explained by the method factor was only 12.3%, well below the 25% cutoff recommended by Podsakoff et al. (2003). This indicates that common method bias is within an acceptable range in this study and does not substantively threaten the findings.

#### 2.3.4 Validation of the compatibility between the measurement model and the overall model

This study conducted a multi-factor confirmatory factor analysis on the measurement models of four latent variables: perceived natural environment, perceived social environment, neighborhood interaction, and older adults’ subjective well-being. The test results showed that the combined reliability (Composite Reliability, CR) of the four latent variables was 0.868, 0.878, 0.849, and 0.857 respectively, all far exceeding the critical standard of 0.7; the average variance extracted (average variance extracted, AVE) was 0.622, 0.643, 0.585, and 0.546 respectively, all higher than the critical standard of 0.5, indicating that the measurement model has good internal consistency and convergent validity (Steenkamp and Maydeu-Olivares, 2023). The standardized factor loadings of all observed variables were above the critical value of 0.6, and the squared multiple correlation coefficients (squared multiple correlation, SMC) were all greater than the 0.36 standard, indicating that the observed variables have good explanatory power for their corresponding latent variables and that the measurement model complies with academic norms (Lai, 2018). In conclusion, the measurement models of all latent variables in this study have good reliability and validity, meeting the prerequisite requirements for structural equation model analysis.

Furthermore, the overall goodness-of-fit test results of the structural equation model in this study are shown in Table 1. All fit indicators met ideal standards: the Root Mean Square Error of Approximation (RMSEA) = 0.048 (95% CI: 0.042–0.054), the Comparative Fit Index (CFI) = 0.961, and the Tucker–Lewis Index (TLI) = 0.954. All these indicators conform to the general evaluation criteria for SEM goodness-of-fit (Shi et al., 2019), indicating that the theoretical model constructed in this study has a good fit with the actual survey data and that the model design is reasonable and empirically supported.

**Table 1:** Comparison of model fit metrics.

Model	TLI	CFI	RMSEA	SRMR
Model data	0.954	0.961	0.048	0.049
Idealized standard	>0.90	>0.90	<0.08	<0.08

## 3 Results

### 3.1 Description of the descriptive statistics of the research variables

Descriptive statistics for all core variables are summarized in Table 2. For the perceived natural environment, the mean scores of individual observed items ranged from 3.102 to 3.503, reflecting an overall moderately positive perceived natural environmental quality among participants. The item measuring perceived air pollution yielded the lowest mean score, demonstrating that perceived air pollution stands out as the most prominent natural environmental concern reported by older adults. By comparison, the item regarding insufficient lighting obtained the highest mean value, implying that older adults perceived lighting shortage as the least severe environmental issue.

**Table 2:** Variable descriptive statistics.

Variable names	Observed variables	Variable items	Mean scores		
			Mean (all)	Mean (male)	Mean (female)
Subjective well-being	WHO-5 scale assessment	Do you feel happy and in a good mood?	3.963	4.135	3.798
		Do you feel peaceful and relaxed?	3.950	4.012	3.891
		Do you feel energetic and full of vitality?	3.429	3.512	3.348
		Did you feel refreshed and well-rested when you woke up?	4.001	4.135	3.870
		Do you think that your daily life is full of interesting things?	3.597	3.756	3.444
Perceived natural environment	Perceived air pollution	The level of air pollution in the community	3.102	3.147	3.058
	Perceived water pollution	The degree of water pollution in the community	3.115	3.151	3.080
	Perceived noise pollution	Noise in the community	3.151	3.194	3.111
	Insufficient lighting	The problem of insufficient lighting in the community	3.503	3.524	3.482
Perceived social environment	Community safety	Does the community where you live give you a sense of security?	4.279	4.270	4.288
	Community convenience	Is it convenient to buy groceries and do shopping in the place where you live?	4.129	4.149	4.109
	Community assistance	When I need help, the community is willing to assist me.	3.868	3.830	3.905
	Community management	Are you satisfied with the management of your community (property/neighborhood)?	3.758	3.827	3.693
Neighborhood interaction	Cultural and recreational activities	The frequency of engaging in cultural and recreational activities with neighbors	3.557	3.507	3.608
	Neighborhood frequency	The frequency of mutual assistance among neighbors within the community	4.013	3.979	4.050
	Chatting gatherings	The frequency of chatting and gathering with neighbors	4.154	3.940	4.357
	Neighbor visits	The frequency of visiting neighbors’ homes	3.639	3.501	3.770
Control variables	Gender	What is your gender?	1.513	–	–
	Age	What is your age?	70.208	70.261	70.159
	Educational attainment	What is your highest educational attainment?	4.115	4.786	3.467
	Marriage	At present, what is your marital status?	1.702	1.764	1.643
	Number of cohabiting individuals	At present, how many people do you live with?	2.250	2.226	2.274
	Self-assessed health	How do you feel about your current health condition?	3.072	3.161	2.986
	Self-perceived economic class	Which economic class do you think you belong to?	4.246	4.243	4.248

In terms of the perceived social environment, item mean scores varied between 3.758 and 4.279. Indicators of community safety and daily shopping convenience both presented mean values above 4.0, which reflected generally favorable perceptions and satisfactory evaluations of neighborhood safety conditions and basic daily service accessibility among surveyed older adults. In contrast, items concerning community management quality and community supportive services showed relatively lower average scores, indicating considerable room for improvement in community governance performance and targeted supportive service provision at the neighborhood level.

For subjective well-being of older adults, the means of the observed indicators of older adults’ subjective well-being ranged from 3.597 to 4.001, indicating a moderately high level of subjective well-being among the surveyed older adults. Items measuring vitality and daily life interest reported the lowest average levels, which indicated diminished physical energy and limited positive daily experiences among older residents. Notably, the item means and total score of subjective well-being were significantly lower among older women than among older men, suggesting a relatively lower level of well-being in the female older adult group.

With regard to neighborhood interaction, the mean values of all items fell between 3.557 and 4.154, corresponding to a moderate overall frequency of daily neighborhood social engagement. Items related to neighborhood mutual assistance and casual daily chatting obtained relatively higher scores, whereas indicators of joint recreational activities and in-home neighbor visiting showed notably lower ratings. This distribution suggests that daily neighborhood interactions among older adults remain limited to superficial, low-intensity social exchanges such as routine communication and practical mutual aid, with less frequent in-depth social engagement and close-knit neighborhood bonding. Additionally, female older adults reported more frequent neighborhood interaction than male participants.

Descriptive results for control variables further illustrated the basic demographic profile of the sample. The gender distribution was relatively balanced, with females accounting for 51.3% of the total respondents. The average age of participants was 70.208 years, ranging from 60 to 95 years. Most older adults had only primary education or below, and male respondents exhibited a higher average educational attainment than females. Approximately 70.2% of participants were currently married, with a larger proportion of married older men than women. Social class was concentrated at the lower-to-middle level, and self-rated physical health remained at a moderate overall level, with males reporting better self-perceived health status than females.

### 3.2 Analysis of the fitting results of the full sample model

**Figure 1:**
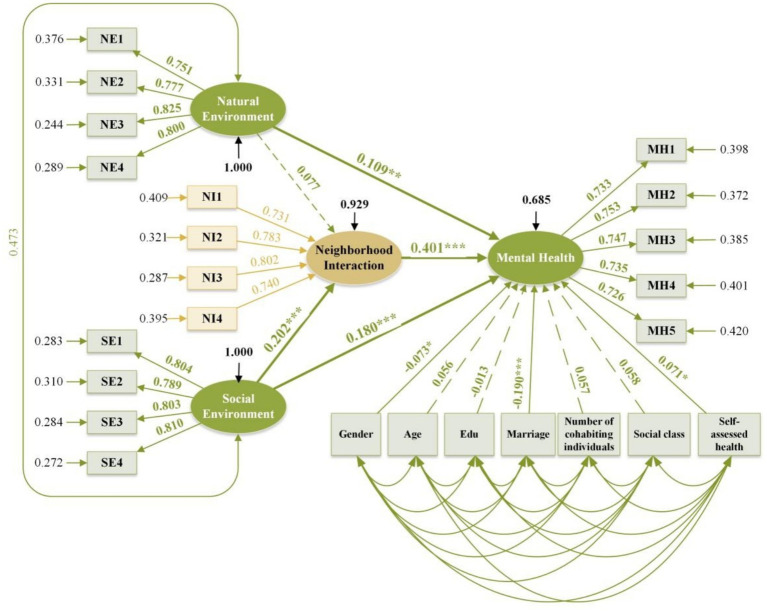
Overall sample model standardisation path diagram.

A SEM was employed to examine the associations between the perceived natural environment, perceived social environment, and older adults’ subjective well-being, as well as the mediating role of neighborhood interaction. After controlling for all demographic and socioeconomic control variables, the decomposition results of core path effects are presented in Table 3, with the effect paths visualized in Figure 1. Specific findings are detailed as follows:

**Table 3:** Model statistical results of the overall sample.

Variables	Independent variables		Mediating variable	Dependent variable: Subjective well-being		
–	–	–	Neighborhood interaction	Overall effect	Direct effect	Indirect effect
Independent variable	Perceived natural environment		0.077	0.140**	0.109**	0.031
	Perceived social environment		0.202***	0.261***	0.180***	0.081***
Mediating variable	Neighborhood interaction		–	0.401***	0.401***	
Control variables	Gender				−0.073*	
	Age				0.056	
	Education				−0.013	
	Marriage				−0.190***	
	Number of cohabiting individuals				0.057	
	Social class				0.058	
	Self-assessed health				0.071*	

***p < 0.001, **p < 0.01, *p < 0.05.

The total association between perceived natural environment and older adults’ subjective well-being was significantly positive [*β* = 0.140, *p* = 0.006, 95% CI (0.102, 0.178)], and the direct association was also significantly positive [*β* = 0.109, *p* = 0.008, 95% CI (0.071, 0.147)]. Bootstrap results showed that the 95% confidence interval for the indirect association via neighborhood interaction included zero [*β* = 0.031, 95% CI (−0.008, 0.068)], indicating that the indirect association was not statistically significant. These findings indicate that perceived natural environment exhibited only a significant direct positive association with older adults’ subjective well-being; no significant indirect association via neighborhood interaction was observed.

The total association between perceived social environment and older adults’ subjective well-being was significantly positive [*β* = 0.261, *p* < 0.001, 95% CI (0.225, 0.297)], and the direct association was significantly positive [*β* = 0.180, *p* < 0.001, 95% CI (0.143, 0.217)]. Bootstrap results indicated that the 95% confidence interval for the indirect association via neighborhood interaction did not contain zero [*β* = 0.081, *p* < 0.001, 95% CI (0.054, 0.109)], confirming a significant indirect association. These results show that perceived social environment was positively associated with older adults’ subjective well-being, with the association comprising both a direct association and an indirect association via neighborhood interaction; neighborhood interaction partially accounted for this association, with the indirect association representing 31.03% of the total association. Overall, compared with perceived natural environment, perceived social environment demonstrated a stronger positive association with older adults’ subjective well-being.

Further examination of the associations with neighborhood interaction revealed that only perceived social environment was significantly positively associated with neighborhood interaction (*β* = 0.202, *p* < 0.001), whereas the association between perceived natural environment and neighborhood interaction was not statistically significant (*p* > 0.05). In addition, neighborhood interaction was significantly positively associated with older adults’ subjective well-being (*β* = 0.401, *p* < 0.001). These findings are fully consistent with the results of the indirect association tests.

Regarding the control variables, sex (*β* = 0.073) and self-rated health (*β* = 0.071) were significantly positively associated with older adults’ subjective well-being, suggesting that being male and reporting better self-rated health corresponded to relatively higher levels of subjective well-being. Marital status (*β* = −0.190) was significantly negatively associated with subjective well-being, suggesting that older adults who were currently married reported relatively higher subjective well-being. These results are consistent with the established findings in environmental health research, further confirming the robustness of the model and the credibility of the conclusions.

### 3.3 Comparison of model results across gender groups

To examine the applicability of the scale among older men and older women, configural, metric, scalar, and structural invariance were sequentially tested (Table 4). The configural invariance model exhibited acceptable fit (CFI = 0.961, RMSEA = 0.048), confirming equivalent factor structures and item assignments across the two groups. The metric invariance test yielded ΔCFI = −0.003 and ΔRMSEA = 0.001 relative to the baseline model, not exceeding the critical thresholds, thus establishing equivalent factor loadings and satisfying the prerequisite for comparing path coefficients between groups. In the scalar invariance test, ΔCFI = −0.004 and ΔRMSEA = 0.001, also meeting the invariance criterion, indicating no group bias in item intercepts and supporting cross-group comparison of latent means. Further testing of structural invariance showed that compared with the scalar invariance model, ΔCFI = −0.014 and ΔRMSEA = 0.006; the ΔCFI value exceeded the cutoff, suggesting marked differences in the core association patterns between older men and older women and warranting in-depth multi-group analysis.

**Table 4:** Results of multi-group measurement invariance testing.

Model	*χ* ^2^	df	CFI	TLI	RMSEA (95% CI)	ΔCFI	ΔRMSEA	Conclusion
Configural invariance	428.76	164	0.961	0.953	0.048 (0.042–0.054)	–	–	Supported
Metric invariance	445.23	176	0.958	0.951	0.049 (0.043–0.055)	−0.003	0.001	Supported
Scalar invariance	472.58	188	0.954	0.948	0.050 (0.044–0.056)	−0.004	0.001	Supported
Structural invariance	521.34	194	0.947	0.942	0.054 (0.048–0.060)	−0.0014	0.006	Not supported

These results indicate that the observed gender differences in the path coefficients are free from measurement bias and reflect genuine structural divergence between the two groups.

**Figure 2:**
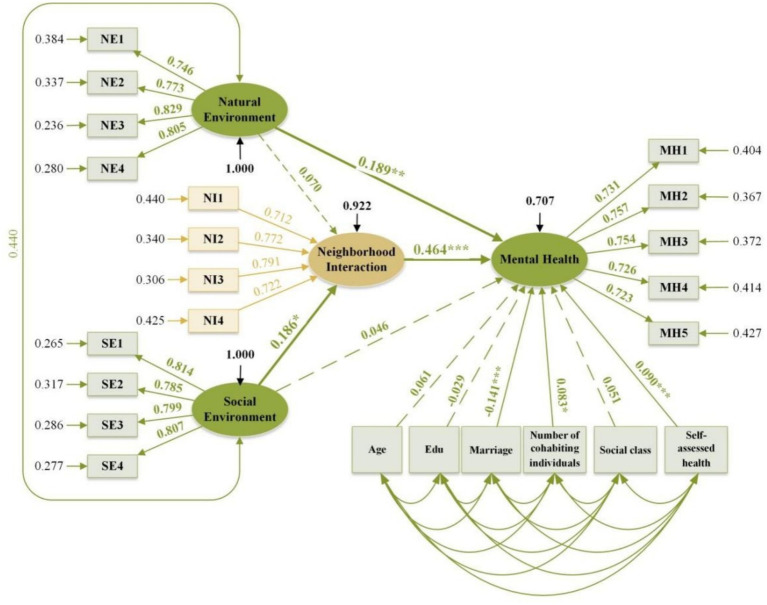
Statistical results of the model for male older adults.

**Figure 3:**
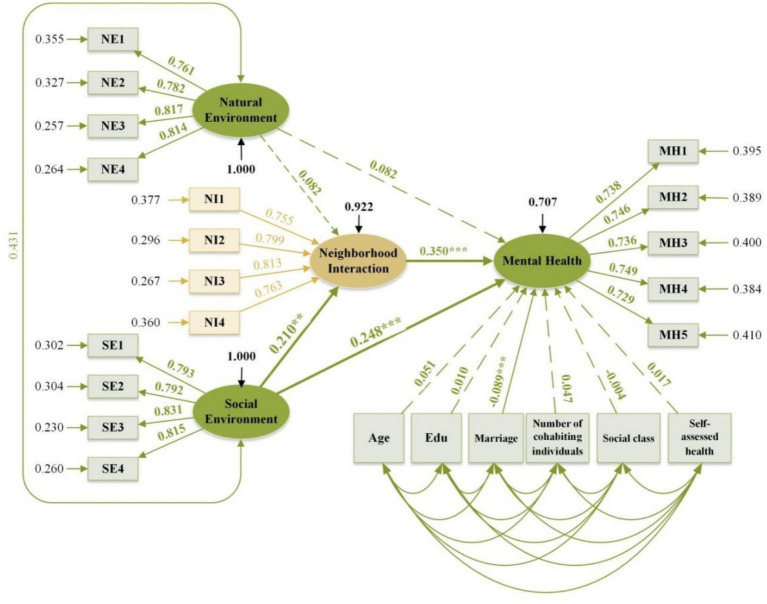
Statistical results of the model for female older adults.

Having established the cross-gender comparability of the measurement model, multi-group structural equation modeling was employed to examine the gender-specific patterns of the “perceived natural environment/perceived social environment—neighborhood interaction—subjective well-being of older adults” associations. Older men (Group 1, *n* = 1,426) and older women (Group 2, *n* = 1,503) were set as comparison groups. Nested model comparisons revealed a significant difference in fit between the unconstrained model and the model with equality constraints on structural coefficients, confirming gender differences in the path coefficients. Table 5, Figures 2, 3 present the model results for the two gender groups.

**Table 5:** Comparison of the model results in different gender groups.

Variables	Independent variables		Mediating variable	Dependent variable: subjective well-being		
Male						
–	–	–	Neighborhood Interaction	Overall effect	Direct effect	Indirect effect
Independent variable	Perceived natural environment		0.070	0.222**	0.189**	0.033
	Perceived social environment		0.186*	0.132	0.046	0.086
Mediating variable	Neighborhood interaction				0.464***	
Control variables	Age				0.061	
	Education				−0.029	
	Marriage				−0.141**	
	Number of cohabiting individuals				0.083*	
	Self-perceived economic class				0.051	
	Self-assessed health				0.090***	
Female						
Independent variable	Perceived natural environment		0.082	0.112	0.082	0.030
	Perceived social environment		0.210**	0.322***	0.248***	0.073**
Mediating variable	Neighborhood interaction				0.350***	
Control variables	Age				0.051	
	Education				0.010	
	Marriage				−0.089*	
	Number of cohabiting individuals				0.047	
	Self-perceived economic class				−0.004	
	Self-assessed health				0.017	

***p < 0.001, **p < 0.01, *p < 0.05.

In the older male group, the total association between perceived natural environment and subjective well-being of older adults was significantly positive (*β* = 0.222, *p* = 0.002), and the direct association was significantly positive (*β* = 0.189, *p* = 0.005); the 95% confidence interval for the indirect association via neighborhood interaction contained zero [*β* = 0.033, 95% CI (−0.012, 0.079)], indicating no significant indirect association. The total, direct, and indirect associations between perceived social environment and older men’s subjective well-being were all non-significant (all *p* > 0.05). These results show that among older men, perceived natural environment exhibited a significant direct positive association with subjective well-being, while no indirect association through neighborhood interaction was observed, and perceived social environment was not significantly associated with older men’s subjective well-being.

In the older female group, the total association between perceived social environment and subjective well-being was significantly positive (*β* = 0.322, *p* < 0.001), and the direct association was significantly positive (*β* = 0.248, *p* < 0.001); the 95% confidence interval for the indirect association via neighborhood interaction did not contain zero [*β* = 0.073, *p* = 0.005, 95% CI (0.041, 0.106)], indicating a significant indirect association. The total, direct, and indirect associations between perceived natural environment and older women’s subjective well-being were all non-significant (all *p* > 0.05). These findings indicate that for older women, only perceived social environment was positively associated with subjective well-being, and this association comprised both a direct and an indirect component through neighborhood interaction, with neighborhood interaction partially accounting for the association.

Furthermore, regarding the associations between perceived environment types and neighborhood interaction, the positive association between perceived social environment and neighborhood interaction was stronger in the female group (*β* = 0.210, *p* < 0.01) than in the male group (*β* = 0.186, *p* < 0.05). The positive association between neighborhood interaction and subjective well-being was stronger in the male group (*β* = 0.464, *p* < 0.001) than in the female group (*β* = 0.350, *p* < 0.001).

### 3.4 Robustness check with multiple imputation

To verify whether the findings obtained with listwise deletion were sensitive to the missing data treatment, a robustness check was conducted using multiple imputation on the full sample. Multiple imputation by chained equations generated 20 complete datasets; structural equation models were fitted with robust maximum likelihood estimation, and the results were combined using Rubin’s rules. The decomposition of the core associations is presented in Table 6.

**Table 6:** Robustness check with multiple imputation.

Path	*β* (MI)	95% CI (MI)	*p*-value (MI)	*β* (listwise deletion)	Consistency of conclusions
Perceived natural environment → Subjective well-being (direct association)	0.107**	[0.069, 0.145]	0.002	0.109**	Highly consistent
Perceived natural environment → neighborhood interaction	0.078	[−0.011, 0.161]	0.088	0.077	Highly consistent
Perceived natural environment → neighborhood interaction → subjective well-being (indirect association)	0.031	[−0.010, 0.068]	0.143	0.031	Consistent
Perceived social environment → subjective well-being (direct association)	0.183***	[0.145, 0.220]	<0.001	0.180***	Highly consistent
Perceived social environment → neighborhood interaction	0.197***	[0.125, 0.273]	<0.001	0.202***	Highly consistent
Perceived social environment → neighborhood interaction → subjective well-being (indirect association)	0.078***	[0.051, 0.105]	<0.001	0.081***	Highly consistent
Neighborhood interaction → subjective well-being	0.394***	[0.329, 0.457]	<0.001	0.401***	Highly consistent

*p < 0.001, **p < 0.01, *p < 0.05.

The multiple imputation results showed that the direct association between perceived natural environment and subjective well-being was significantly positive [*β* = 0.107, *p* < 0.01, 95% CI (0.069, 0.145)], whereas the indirect association was non-significant [*β* = 0.031, 95% CI (−0.010, 0.068)]. For perceived social environment, both the direct association [*β* = 0.183, *p* < 0.001, 95% CI (0.145, 0.220)] and the indirect association [*β* = 0.078, *p* < 0.001, 95% CI (0.051, 0.105)] were significant. The pattern of neighborhood interaction’s role in linking environmental perceptions and well-being was identical to that obtained with listwise deletion. The direction, magnitude, and statistical significance of all core coefficients were highly consistent with the primary analysis, with no change in conclusions.

In sum, the multiple imputation robustness check confirms that the study’s conclusions are not contingent upon the missing data handling method, and the results based on listwise deletion are highly reliable and replicable.

## 4 Discussion

Drawing on data from the 2021 Chinese General Social Survey (CGSS), this study systematically compared the complex associations among perceived natural environment, perceived social environment, neighborhood interaction, and older adults’ subjective well-being. The overall findings indicate that the total association of perceived social environment with subjective well-being is significantly stronger than that of perceived natural environment; neighborhood interaction shows a significant partial indirect association in the link between perceived social environment and subjective well-being, whereas perceived natural environment mainly exhibits a direct association. These conclusions remain stable across multiple robustness checks, extending the theoretical boundaries of environmental psychology research in a non-Western context with older adult samples and providing empirical evidence for age-friendly community design in China.

The core finding that the association is stronger for perceived social environment than for perceived natural environment may be understood in light of the highly circumscribed daily activity space of Chinese older adults within their neighborhoods. Over 90% of urban older adults’ daily activities are concentrated within the neighborhood scale, and rural older adults’ activity space is even more confined to the village (Bird et al., 2018); accordingly, perceived neighborhood safety, convenient services, and neighborhood support constitute the baseline of their daily lives (Lin et al., 2024). In developed Western countries, where air quality generally meets established standards, the association between the natural environment and well-being may have entered a “well-being enhancement” phase (Bhopal et al., 2025); however, over 30% of Chinese cities still do not meet the national Class II ambient air quality standards, and neighborhood-level pollution exposure remains a prevalent stressor (Wang et al., 2022). This study also found that older adults rated air pollution as the most severe perceived environmental problem. In this context, the association between the natural environment and older adults’ well-being in China mainly takes the form of reduced pollution-related harm rather than health gain, and the total association is therefore weaker than that of the social environment. This finding directly addresses the misconception in the renovation of old residential communities that prioritizes physical infrastructure over the social environment, providing empirical evidence for optimizing the priorities of community intervention.

Furthermore, perceived social environment is not only directly associated with subjective well-being but also exhibits an indirect association through neighborhood interaction, whereas perceived natural environment is only directly associated with subjective well-being. This divergence reflects different patterns of association: at the level of perceived social environment, favorable evaluations are correlated with lower psychological costs of engaging in daily social interactions, making mutual help, group activities, and neighborly visiting more likely to occur (Zhong et al., 2025); these interactions, in turn, are correlated with emotional companionship, information exchange, and a sense of social fulfillment (Wang et al., 2025). At the level of perceived natural environment, air pollution, water pollution, noise pollution, and other factors jointly constitute older adults’ fundamental evaluation of the livability of their residential area. For older adults with declining physical function and a reduced activity radius, better perceived natural environment may be directly correlated with greater daily comfort and restorative experiences, forming a direct association with subjective well-being.

The most innovative finding of this study is that the associations between environmental perceptions and subjective well-being exhibit pronounced gender heterogeneity: perceived natural environment is directly associated with subjective well-being only among older men; perceived social environment is significantly associated with subjective well-being only among older women, and this association includes a partial indirect association through neighborhood interaction. In the Chinese context, this may be attributable to three considerations. First, daily activity patterns differ by gender. Older men’s contact with nature often takes the form of purposeful solitude, directly forming a person–nature connection without reliance on social mediation (Zhang et al., 2005). Second, spatial dependence differs by gender. Shaped by the traditional gender division of labor in China, older women’s daily activities center heavily on family caregiving, confining their activity radius to the neighborhood; consequently, their dependence on community convenience, safety, and mutual assistance is far greater than that of men (Luo, 2025). Third, social support networks differ by gender. Social support for older Chinese women is more dependent on neighborhood-based geographical ties—particularly for widowed or solo-living women, for whom neighborhood interaction serves as a core source of emotional and practical help—whereas men rely more on family, coworkers, and friends (Wu et al., 2019; Xu et al., 2025). This finding challenges the one-size-fits-all design paradigm for age-friendly communities and provides an empirical basis for gender-differentiated community environmental design.

Based on the above findings, this study proposes a shift from universal strategies to precision-oriented community environmental design. First, for older Chinese men, providing a natural environment with less pollution and more daylight represents a more targeted and pertinent approach. In such community design, certain non-interactive open spaces should be retained, incorporating resting areas suitable for solitude. Second, for older Chinese women, greater emphasis should be placed on safe, mutual-help semi-public interaction spaces and the continuous provision of low-barrier community activities. By enhancing perceived neighborhood safety, health promotion, mutual-help elderly care, and other neighborhood services, neighborhood interaction and social support can be strengthened, which in turn is associated with higher subjective well-being among older women. It is noteworthy that the above suggestions should be regarded as planning implications derived from cross-sectional associations rather than confirmed causal intervention outcomes.

This study has three main limitations. First, owing to the cross-sectional design, the study can only identify associations among perceived natural environment, perceived social environment, and older adults’ subjective well-being, but cannot establish a causal relationship between environmental perceptions and subjective well-being. Future research could employ nationally representative longitudinal data and use longitudinal designs to test the causal effect of environmental perceptions on subjective well-being. Second, all core variables were derived from self-reports and may be subject to common method bias. Although the latent method factor model indicated that the average method variance fell within an acceptable range and the main direct associations remained significant after controlling for the method factor, self-report bias cannot be completely ruled out. Future studies could integrate GIS-based spatial analysis techniques and incorporate objective environmental indicators to examine how the interplay of subjective and objective environments is associated with older adults’ subjective well-being, thereby further reducing bias attributable to common method variance. Third, the CGSS data contained a certain proportion of missing values. Although multiple imputation verified the robustness of the findings, further clarification is needed regarding the potential implications of these missing values for sample representativeness and statistical power. In addition, the sample did not cover provinces such as Xinjiang, Tibet, and Hainan. Future research should broaden the sample coverage, attend to the environmental health needs of older populations in border and rural areas, and refine heterogeneity analyses across different regions and urban–rural types.

## 5 Conclusion

The results show that the positive association between perceived social environment and older adults’ subjective well-being is significantly stronger than that of perceived natural environment. The associations of the two types of environmental perception with subjective well-being differ fundamentally: perceived natural environment exhibits only a direct positive association with subjective well-being, whereas perceived social environment is associated with subjective well-being through both a direct positive association and a partial indirect association via neighborhood interaction. This study further reveals pronounced gender heterogeneity in the associations between the two types of environmental perception and older adults’ subjective well-being. Perceived natural environment is significantly and positively associated with subjective well-being only among older men, and this association is characterized exclusively by a direct association. Perceived social environment is significantly and positively associated with subjective well-being only among older women, and this association also features a significant partial indirect association through neighborhood interaction. Drawing on these association findings, age-friendly community design in China should give full consideration to the differentiated environmental needs of older men and older women and establish a gender-sensitive community environmental support planning. For older men, priority should be given to enhancing natural environment elements that are significantly and positively associated with their subjective well-being. For older women, community environment enhancement should be integrated with the provision of neighborhood social spaces to create supportive conditions for neighborhood interaction. This study provides important empirical evidence for the differentiated and refined design of age-friendly communities.

## Data Availability

The original contributions presented in the study are included in the article/supplementary material, further inquiries can be directed to the corresponding authors.
